# Supplementary material for: Deep Learning Model Based on Contrast-Enhanced Computed Tomography Imaging to Predict Postoperative Early Recurrence after the Curative Resection of a Solitary Hepatocellular Carcinoma
Source: Cancers (Basel). 2023 Apr 4;15(7):2140. doi: 10.3390/cancers15072140 (PMC10092973; doi:10.3390/cancers15072140)
Supplement: Supplementary file 1 [file cancers-15-02140-s001.zip › cancers-2245670-supplementary.pdf]

Supplementary figure

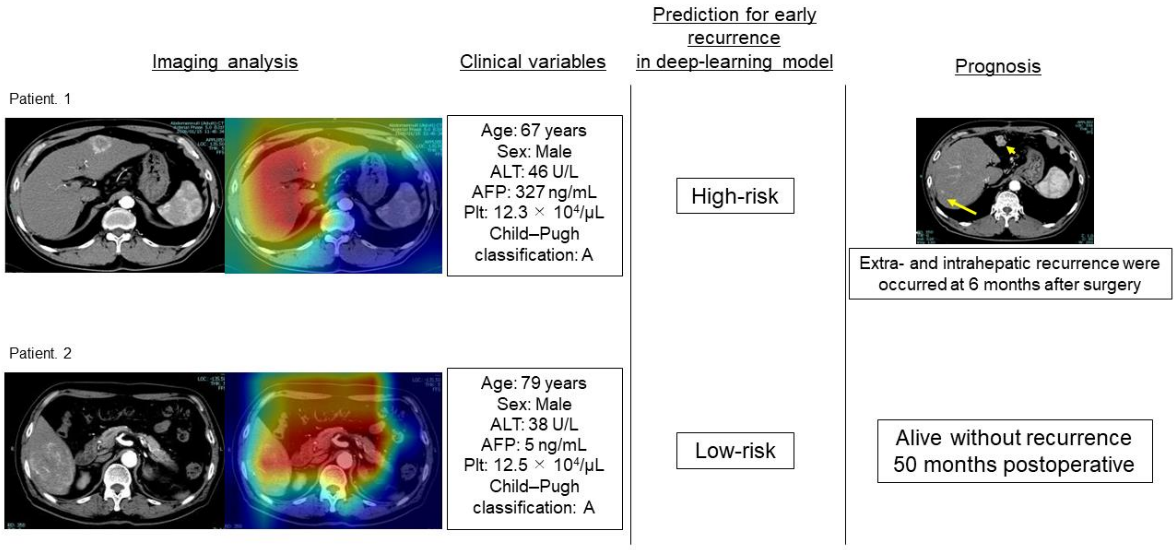

Figure S1. Preoperative clinical variables, including saliency maps and corresponding postoperative prognosis of two patients. In Patient 1 who was categorized into the high-risk group based on the DL model, intrahepatic recurrence (arrow) and peritoneal dissemination (short arrow) were detected in contrast-enhanced computed tomography at 6 months postoperative. Meanwhile, Patient 2, who was categorized into the low-risk group, has achieved recurrence-free survival for >2 years.
